# Squamous cell carcinoma of the tongue with cardiac metastasis on ^18^F-FDG PET/CT: A case report and literature review

**DOI:** 10.1097/MD.0000000000025529

**Published:** 2021-04-16

**Authors:** Pierre Delabie, Diane Evrard, Ilyass Zouhry, Phalla Ou, François Rouzet, Khadija Benali, Eve Piekarski

**Affiliations:** a Department of Nuclear Medicine, Centre Hospitalier Universitaire Bichat; b Department of Otorhinolaryngology and Head and Neck Surgery; c Department of Pathology; d Department of Radiology, Centre Hospitalier Universitaire Bichat, Assistance Publique – Hôpitaux de Paris, Inserm 1148, Université de Paris, Paris, France.

**Keywords:** ^18^F-FDG PET/CT, cardiac metastasis, case report, oncology, tongue malignancy

## Abstract

**Introduction:**

The most common malignancies metastasizing to the heart are cancers of the lung, breast, mesothelioma, melanoma, leukemia, and lymphoma. Cardiac metastasis from a tongue cancer is a rare finding and only a few cases have been reported previously in the literature. In this case report and literature review, we discuss the main clinical features of patients with cardiac metastases secondary to a tongue cancer and imaging modalities performed, especially the ^18^F-Fluorodeoxyglucose positron emission tomography/computed tomography (^18^F-FDG PET/CT).

**Patient concerns:**

This is a case of a 39-year-old woman who in April 2018 was diagnosed with an invasive well differentiated squamous cell carcinoma of the movable tongue. She underwent a left hemiglossectomy followed by a revision of hemiglossectomy and ipsilateral selective neck lymph nodes dissection levels II to III because of pathological margins. An early inoperable clinical recurrence was diagnosed and she received radiochemotherapy with good clinical and metabolic response. She remained asymptomatic thereafter.

**Diagnosis:**

In January 2020, a pre-scheduled ^18^F-FDG PET/CT showed a diffuse cardiac involvement. In February 2020, a biopsy of the lesion revealed a metastatic squamous cell carcinoma.

**Interventions:**

She was deemed to not be a cardiac surgical candidate and treated by palliative chemotherapy: taxol-carboplatin associated with cetuximab then cetuximab alone because of adverse effects. A re-evaluation imaging performed in April 2020 evidenced a progression of the cardiac involvement, which led to switch chemotherapy by immunotherapy with nivolumab.

**Outcomes:**

This patient had a very poor prognosis and succumbed to major heart failure 4 months after the diagnosis of cardiac metastasis.

**Conclusion:**

In this case report, ^18^F-FDG PET/CT proved to be useful in detecting cardiac metastasis and changed the therapeutic management of the patient. It suggests that patients with tongue malignancies in a context of poor initial prognosis should be followed-up early by ^18^F-FDG PET/CT with HFLC diet to facilitate detection of recurrence.

## 1 Introduction

Oral cancer is the 6th most common cancer in the world,^[1]^ with 9 out of 10 being oral squamous cell carcinomas (OSCCs).^[2,3]^ The most important risk factors for OSCCs are use of tobacco and the regular drinking of alcoholic beverages. In addition, infection with high-risk human papillomavirus (HPV) genotypes is associated with the aetiopathogenesis of OSCCs.^[4]^ These cancers are rare before age 40, but the frequency in this age group tends to increase. One of the commonest sites of OSCCs is tongue with 25% to 40% of the occurrences.^[5]^ Compared to other OSCCs, metastatic spread of tongue cancer is facilitated by its rich lymphatic network resulting in an adverse impact on prognosis.^[5]^ Secondary involvement of the heart appears to be extremely rare. There are only very few reports of this atypical complication in the literature because patients are often asymptomatic.

We present here the case of a young female diagnosed with squamous cell carcinoma of the tongue which recurrence has been principally revealed by cardiac metastasis.

## 2 Case presentation

A 39-year-old woman with a 3 pack-year smoking history was initially diagnosed in April 2018 with an invasive well-differentiated squamous cell carcinoma of the movable tongue. An initial staging ^18^F-Fluorodeoxyglucose positron emission tomography/computed tomography (^18^F-FDG PET/CT) showed an isolated moderately hypermetabolic focus on the left side of the tongue (SUV_max_ 6.2). She underwent a left hemiglossectomy in May 2018 followed by a revision of hemiglossectomy and ipsilateral selective neck lymph nodes dissection levels II to III in October 2018 because of pathological margins. The final histological results were persistent in situ carcinoma in the tongue with complete resection and metastatic carcinoma in one lymph node on the 40 analyzed lymph nodes. Therefore, this tongue carcinoma was finally classified pT2pN1M0 and no adjuvant therapy was undertaken.

Two months later, an early clinical recurrence was diagnosed following severe tongue pain. A restaging ^18^F-FDG PET/CT showed an intensely hypermetabolic focus along the left side of the tongue (SUV_max_ 16.6) and a mildly hypermetabolic left retropharyngeal lymph node (SUV_max_ 4.0) with no evidence of distant metastasis. The patient received chemotherapy by taxol-carboplatin then cisplatin followed by external radiochemotherapy from January 2019 to March 2019 with good clinical and metabolic response. She remained asymptomatic thereafter.

**Figure 1:**
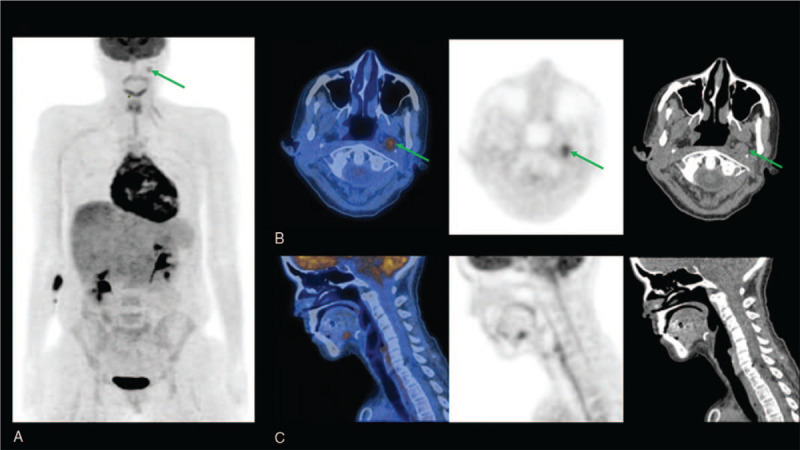
(A) Anterior view of 3D Maximum intensity projection PET image showing an intense and pathological FDG uptake in the basal posterolateral wall of the right ventricle and in the pericardium with a low FDG uptake in one left retropharyngeal lymph node consistent with metastasis (green arrow). (B) Axial PET/CT, PET, and CT images of the nasopharynx showing the pathological left retropharyngeal lymph node (green arrow). (C) Sagittal PET/CT, PET, and CT images showing no tongue abnormality.

**Figure 2:**
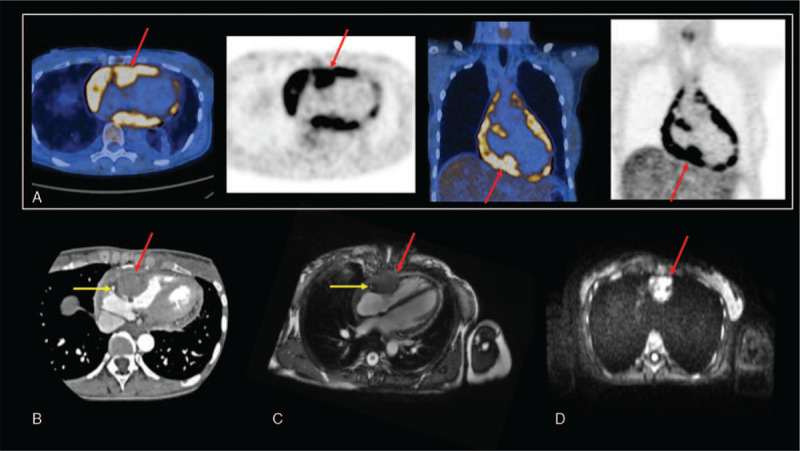
(A) Axial and coronal fused PET/CT and PET images of the thorax showing an intense and pathological FDG uptake in the basal posterolateral wall of the right ventricle (red arrow) and in the pericardium. (B) Cardiac CT scan image shows the cardiac metastasis infiltrating the basal posterolateral wall of RV (red arrow), in front of the right coronary artery (yellow arrow) with a thickening of the entire pericardium. Cardiac magnetic resonance imaging. (C) 2D-SSFP (FIESTA) image four-chamber view shows a 4.6 × 3.6 cm mass infiltrating the basal posterolateral wall of RV (red arrow), in front of the right coronary artery (yellow arrow), extended to the basal posterolateral wall of right atrium. (D) Diffusion-weighted imaging at *b* = 1000 s/mm^2^ view shows an area of increased signal corresponding with the mass of RV (red arrow).

**Figure 3:**
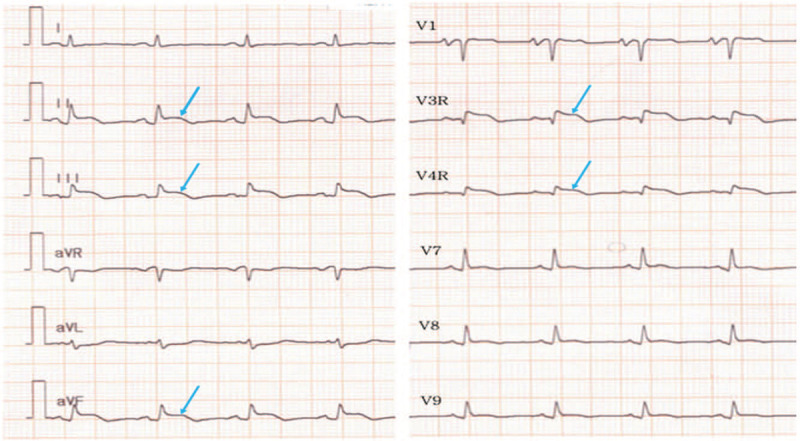
Electrocardiogram shows ST elevation in the inferior and right leads (blue arrows) with T-wave inversions.

In January 2020, a pre-planned ^18^F-FDG PET/CT, with High-Fat and Low-Carbohydrate (HFLC) dietary preparation included to explore electrocardiogram (ECG) abnormalities of recent onset, revealed an intensely hypermetabolic myocardial focus (SUV_max_ 7.1) in the basal posterolateral wall of the right ventricle (RV) associated with diffuse and hypermetabolic pericardial thickening (SUV_max_ 5.6) and a low intensity pathologic hypermetabolic left retropharyngeal lymph node (SUV_max_ 2,5) (Figs. 1 A–C and 2A). The ECG showed ST elevation in V3, the inferior (II, III, VF) and right (V3R, V4R) leads with T-wave inversion (Fig. 3). Further diagnostic imaging with transthoracic echocardiography (TTE) and cardiac CT scan were performed. Cardiac CT scan revealed a 4.6 × 3.5 cm size mass infiltrating the basal posterolateral wall of RV, extended to the right atrium with a thickening of the entire pericardium and a moderate infiltration of the 1st segment of the right coronary artery (Fig. 2.B).

To further characterize the lesion, cardiac magnetic resonance imaging (CMR) confirmed the tissular mass infiltrating the basal posterolateral wall of RV extended to the basal posterolateral wall of right atrium (Fig. 2C), a tissue infiltration encasing the ascending aorta and a thickening of the entire pericardium. There was a high pathological enhancement after contrast agent injection and a high signal on diffusion-weighted imaging of this features (Fig. 2D).

**Figure 4:**
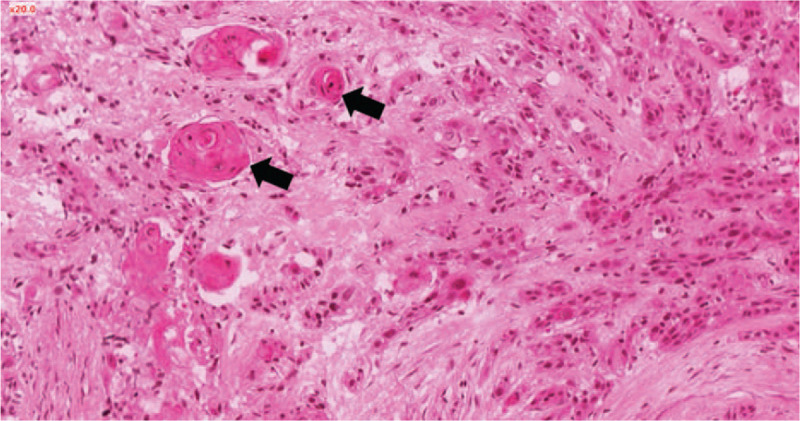
Histological specimen (hematoxylin and eosin stain, original magnification ×200) showing pericardium massively infiltrated by invasive squamous cell carcinoma with typical features including irregular nests, stroma reaction and keratin pearl formation (black arrows).

Cardiac metastasis secondary to squamous cell carcinoma of the tongue was suspected. Nevertheless, because of the atypical presentation, the patient was referred for a surgical biopsy of the pericardium in February 2020 to eliminate a primary cardiac tumor. Pathologic specimen analysis revealed a squamous cell carcinoma metastasis with typical features including irregular nests, stroma reaction and keratin pearl formation (Fig. 4). Immunohistochemistry tests showed a low expression of PD-L1 (<1%) and no expression of the HPV 16.

The location and extent of the cardiac metastasis precluded surgical resection. Thus, a chemotherapy by taxol-carboplatin associated with cetuximab was initiated, then by cetuximab alone because of common side effects due to taxol-carboplatin. Follow-up TTE evidenced in April 2020 a progression of the cardiac involvement with a metastatic invasion of pulmonary trunk and a compression of the right ventricular outflow tract. A systemic immunotherapy by nivolumab was then introduced but the patient died early in May 2020, after 2 cycles of treatment.

## 3 Discussion

Although oral cancers are relatively common with respect to all diagnosed malignancies, very few cases of ante-mortem diagnosed cardiac metastasis of OSCCs have been reported in the literature, possibly because of a lack of systematic whole-body follow-up imaging. In a review of the literature from the year 1985 to October 2019, we found 16 other cases of patients with squamous cell carcinoma of the tongue and cardiac metastasis (Table 1).

**Table 1:** Comparison of characteristics between cases with squamous cell carcinoma of the tongue metastasizing to the heart.

Reference	Sexe	Age (yr)	Primary treatment	Delay (mo)	Cardiac Metastasis	Metastasis	Symptoms	ECG Findings	Initial Imaging Modality	Treatment/Survival time
Werbel et al^[19]^	F	61	Hemiglossectomy	18	Cardiac mass located essentially intrapericardially	Bones	Intermittent positionnal chest pain, dysphagia, weight loss	ST depression with T-wave inversions anteriorly	2D Echocardiogram	Planned to proceed with radiotherapy but patient expired before initiation/7 wks
Rivkin et al^[20]^	M	57	Local excision and adjuvant radiotherapy to primary site and bilateral neck	3	Right ventricle	Mediastinal nodes	Chest pain, lower extremity edema	Atrial fibrillation with ST elevation in V2 to V6	Chest X-ray and Echocardiogram	Chemotherapy with cisplatin, 5-FU, bleomycin and methotrexate
Shimoyama et al^[21]^	M	71	Partial glossectomy	10	Left ventricle	Multimetastatic	None	ST elevation in I, VL, V5-6 and ST depression in II, III, VF and V1-3	Echocardiogram	Radiotherapy and chemotherapy/4 wks
Hans et al^[22]^	M	54	Induction chemotherapy (5-FU/cisplatin), glossectomy and left radical neck dissection and adjuvant radiotherapy to primary site and neck to 60 Gy/46 Gy	10	Right ventricle extending into pulmonary infundibulum	No	Dyspnea, lower extremity edema, hemoptysis	Right bundle branch block	CT Chest	Supportive care
Nagata et al^[23]^	M	59	Preoperative concurrent chemoradiation therapy to 30 Gy followed by partial glossectomy and right radical neck dissection and rectus abdominis musculocutaneous flap reconstruction followed by adjuvant chemotherapy	17	Left atrium to the left pulmonary vein, pericardium	No	Fever	N/A	CT Chest and Echocardiogram	Resection of cardiac mass/3 weeks
Onwuchekwa and Banchs^[24]^	F	45	Right partial glossectomy and extensive neck dissection	17	Right ventricle invading interventricular septum and left ventricle	Multimetastatic	Syncope, mild dyspnea	Sinus rythm	CT angiogram and 2D echocardiogram	Supportive care
	F	36	Concurrent chemoradiotherapy, left partial glossectomy, left neck dissection	18	Anteroseptal wall of the left ventricle extending toward the right ventricular outflow tract, pericardial effusion	Multimetastatic	Palpitations, dyspnea	ST elevation in the anterolateral leads	Chest X-ray and 2D echocardiogram	Radiotherapy and chemotherapy/8 wks
Yadav et al^[25]^	M	76	Partial glossectomy	120	Left and right ventricle with extension to chordae tendinae	Multimetastatic	None	ST elevation in the anterolateral leads	Chest X-ray and Echocardiogram	Supportive care/4 wks
Puranik et al^[26]^	F	32	Wide excision and right lateral neck dissection	24	Left ventricle	Lung	None	N/A	PET/CT	Palliative chemotherapy
Browning et al.^[27]^	M	50	Radiotherapy followed by total glossectomy and bilateral neck dissections	9	Anterior wall of right ventricle	No	None	N/A	PET/CT	Supportive care
Malekzadeh et al^[28]^	F	58	Right hemiglossectomy and adjuvant radiotherapy	132	Right ventricle	Multimetastatic	Acute chest pain	Slight ST elevation in V3 and V4	CT Chest	Palliative chemotherapy with cetuximab, carboplatin and 5-FU/7 wks
Chua et al ^[29]^	M	63	Resection and reconstruction	60	Right ventricle	No	Progressive dyspnea	N/A	Echocardiogram	Concurrent chemoradiotherapy
Kim et al^[8]^	F	46	Left hemiglossectomy and bilateral neck dissection	36	Left ventricle	Multimetastatic	Chest pressure, dizziness, dyspnea	T-waves inversion in the inferior and V3-V6 leads	CT Chest	Palliative immunotherapy with nivolumab
Tandon et al^[30]^	F	25	Hemiglossectomy	16	Left and right ventricle	Multimetastatic	Dyspnea on exertion	ST elevation in the inferior leads and T-wave inversions in the anterolateral leads	Echocardiogram	Supportive care
Nanda et al^[31]^	M	47	N/A	N/A	Right ventricle, pericardium	Multimetastatic	Severe dizziness, chest tightness, dyspnea, nights sweats, left upper back pain	Diffuse ST elevation	PET/CT	Palliative immunotherapy with nivolumab
Shafiq et al.^[32]^	M	43	Tracheostomy, right neck dissection, right tongue cancer resection and reconstruction with a free flap graft from forearm	24	Left ventricular apex	Lung	None	ST elevation in the anterior and lateral leads	CT scan	Palliative immunotherapy with pembrolizumab then chemotherapy with 5-FU, carboplatin and cetuximab
Present Study	F	39	Left hemiglossectomy and ipsilateral selective neck lymph nodes dissection levels II-III	21	Right ventricle with extension to right atrium, pericardium	No	None	ST elevation in V3, the inferior and right leads	PET/CT	Palliative chemotherapy with taxol-carboplatin and cetuximab then immunotherapy with nivolumab/16 wks

Cardiac metastases are far more common than primary cardiac tumors and the most common malignancies spreading to the heart are cancers of the lung, breast, mesothelioma, melanoma, leukemia, and lymphoma.^[6]^ Among head and neck cancers associated with cardiac metastasis at autopsy, tongue cancer is the most frequent primary location, accounting for one-ninth (∼11%) of patients.^[7]^ In a literature review of cardiac metastasis of head and neck cancer detected ante-mortem, Kim et al reported 23 cases of which 12 were tumors of the tongue thus representing a large majority with 52%.^[8]^

The structure of the lymphatic system in the heart may explain the relatively low incidence of cardiac metastases compared with other organs in OSCCs. Cardiac metastases are located, by decreasing order of frequency, to the pericardium, myocardium, epicardium, endocardium, and intracavitary regions.^[9]^ Pericardial effusion has been identified as a direct sign of cardiac metastasis, sometimes presenting as the first manifestation.^[6]^ In this literature review, we observed a pericardial involvement in 29% of patients, a left atrial involvement in one case and a myocardial involvement in most cases. Metastases were located in the right and left ventricles, respectively, in about 60% and 40% of cases. Myocardial involvement is almost exclusively the result of retrograde lymphatic spread through tracheal or bronchomediastinal channels.^[6]^

Table 1 shows an unexpected high proportion of women with squamous cell carcinoma of the tongue (47% of cases), while the overall incidence of OSCCs is twice greater in males than in women^[10]^ and an average of 51 ± 14 years, consistent with other oropharyngeal cancer demographic reports in general. Mean duration between diagnosis of tongue cancer and cardiac metastasis was relatively short at 20 ± 14 months, after exclusion of two patients with extreme intervals of 120 months or more. Although cardiac metastasis usually occurred in patients with advanced stages of the disease, this location was isolated in almost a third of them.

Since symptoms in this location are either absent (6/17 symptomatic cases in our study) or nonspecific, cardiac metastasis is difficult to diagnose and usually detected in the postmortem setting at autopsy. ECG may have a diagnostic value, but generally consists of repolarization abnormalities which are poorly specific. ST segment changes were reported in some patients and the persistence of ST elevation without Q waves has been suggested to be pathognomonic of tumor invasion of the myocardium.^[11]^ When patients were symptomatic or in case of ECG abnormalities, TTE was the most frequent imaging modality used owing to its availability and sensibility for detection of cardiac metastasis.^[12]^ Follow-up CT scan often are done in these patients and may help unmask such rare cardiac involvement. Because of its excellent contrast in soft tissues, CMR was used to characterize the malignant-suspected cardiac masses and the surrounding extension in a few of the reported cases (5/16). In the present literature review and with this case report, ^18^F-FDG PET/CT was the initial imaging modality in 24% of the patients revealing cardiac metastasis from a tongue cancer. ^18^F-FDG PET/CT can be proposed as an option in the monitoring of SCC of the head and neck for the detection of occult recurrence.^[13]^ Some authors have suggested that ^18^F-FDG PET/CT yields good diagnostic performance in long-term surveillance and imparts added value to clinical assessment.^[14,15]^ To improve the diagnostic performance of ^18^F-FDG PET/CT and suppress the physiological myocardial uptake, a HFLC diet followed by 12-h fasting must be applied.^[16]^

Treatment options for patients with cardiac metastasis are limited. Due to the extent of the cardiac involvement, the patient presented here was not eligible for surgical resection. Given the lack of evidence, the role for chemotherapy or radiation remains undetermined.^[17]^ Ferris et al suggested that a PD-L1 expression level of 1% or more tended to be associated with a better overall survival.^[18]^ In this case report, PD-L1 expression was determined by using Combined Positive Score (CPS), which is the number of PD-L1 staining cells (tumor cells, lymphocytes, and macrophages) divided by the total number of viable tumor cells, multiplied by 100. The CPS was 9.9. Because of progression of the disease on a platinum chemotherapy, an immunotherapy by nivolumab was finally introduced. Nivolumab is a fully human IgG4 anti–PD-1 monoclonal antibody approved by the Food and Drug Administration (FDA) and the European Medical Agency (EMA) for the treatment of platinum-resistant recurrent and/or metastatic SCC of the head and neck.

Metastatic involvement of the heart from tongue cancer is an uncommon finding. The diagnosis is often difficult and delayed, as symptoms and signs are absent or nonspecific. In the patient presented here, ^18^F-FDG PET/CT proved to be useful in detecting cardiac metastasis and changed the therapeutic management from potentially curative intent radical salvage therapy to palliative care. ^18^F-FDG PET/CT is a highly sensitive technique, which provides the unique advantage of scanning the whole body thus reducing the risk of missing distant metastasis with a high negative predictive value.^[14,15]^

This case report suggests that patients with tongue malignancies in a context of poor initial prognosis should be followed-up by ^18^F-FDG PET/CT with HFLC diet to facilitate early detection of recurrence, especially in heart, and guide optimal therapeutic management.

## Acknowledgments

We would like to thank Dr Khaldoun KERROU for kindly reading and reviewing the manuscript and for his enriched discussion.

## Author contributions

**Conceptualization:** Pierre DELABIE.

**Supervision:** François ROUZET.

**Writing – original draft:** Pierre DELABIE, Ilyass ZOUHRY.

**Writing – review & editing:** Diane EVRARD, Phalla OU, François ROUZET, Khadija BEN ALI, Eve PIEKARSKI.
